# Sufficient sleep quality easily measured: a multicenter centre study in dutch ICUS

**DOI:** 10.1186/2197-425X-3-S1-A725

**Published:** 2015-10-01

**Authors:** P Rood, P Pickkers, JG van der Hoeven, M van den Boogaard

**Affiliations:** Radboud University Medical Center, Intensive Care Medicine, Nijmegen, Netherlands

## Introduction

Sleep is a fundamental need for recovery while a lack of good sleep is associated with adverse effects. ICU patients have an increased risk for disturbed sleep. Various sleeping questionnaires have been developed to assess the different aspects of sleep. The Richards Campbell Sleep Questionnaire (RCSQ) is one of the most commonly used sleep assessment tools for the ICU consisting of 5 questions on different aspects of sleep and reasons for poor sleep. However, it seems irrelevant to burden patients that state to have a good night sleep with the complete RCSQ. For clinical and developmental use it is useful to quantify sleep in a simple and effective manner. Therefore, the aim of our study was to investigate a simple sleeping numeric rating score; NRS sleep.

## Objectives

To determine a cut-off value for sufficient sleep using a simple numeric rating score for sleep (NRS) to assess the patient perceived quality of sufficient sleep. Secondly, to get insight in de the perceived quality of sleep and the source of sleeping problems in ICUs in the Netherlands.

## Methods

A prospective multicentre cohort study was performed in 19 Dutch ICUs. All centres were visited twice. ICU patients who were able to communicate and were admitted for at least one full night were included. Patients were asked to rank their perceived sleep of the last night on a scale of 0-10, if this sleep quality was sufficient, and subsequently the RCSQ was performed. Logistic regression analysis and the AUROC was used in order to determine the a cut-off for good sleep.

## Results

A total of 468 ICU patients were visited of which 183 patients fully completed the sleeping scores questionnaire. In 194 sleep measurements patients rated their sleep in terms of adequacy, 103 (53%) as sufficient, and 91 (47%) as insufficient. An optimal cut-off value for good sleep was determined at a NRS >5 (table). At the cut-off value the AUROC was 0.81 (95%CI: 0.74-0.87) with a sensitivity of 83% and a specificity of 79%. Pain and noise were the most common reasons given for lack of sleep.

## Conclusions

The cut-off value for adequate sleep is a NRS >5. For clinical practice this may indicate in case of this NRS >5 that the RCSQ is redundant. Only half of the ICU patients experienced a sufficient night sleep. Pain was the most common reason for sleeping problems, followed by noise.

**Table 1:** Performance at different cut-off levels.

NRS	Sensitivity	Specificity	PPV	NPV
>1	1.0	0.24	0.65	0
>2	0.99	0.26	0.65	0.94
>3	0.96	0.39	0.68	0.91
>4	0.93	0.54	0.72	0.85
>5	0.83	0.79	0.84	0.76
>6	0.67	0.92	0.95	0.68
>7	0.46	0.94	0.95	0.56
>8	0.12	0.96	0.9	0.44
>9	0.03	0.98	1	0.42

